# How and why does it work? A video-based qualitative analysis of case conferences to reduce BPSD through the lens of Habermas’s theory of communicative action

**DOI:** 10.1186/s12888-024-05959-x

**Published:** 2024-07-22

**Authors:** Janne Myhre, Bjørn Lichtwarck

**Affiliations:** 1 The Research Centre for Age-Related Functional Decline and Disease (AFS), Innlandet Hospital Trust, Ottestad, Norway; 2 Department of Health and Nursing Science, Faculty of Social and Health Sciences, Inland Norway University of Applied Sciences, INN University, Elverum, Norway; 3 Norwegian Centre for Violence and Traumatic Stress Studies, (NKVTS), Oslo, Norway

**Keywords:** TIME, Case conferences, Video-based study, Communicative actions, Habermas, BPSD

## Abstract

**Background:**

Case conferences are described as a goal-oriented, systematic method that team members can use to exchange professional opinions and develop treatment actions for a particular care problem. However, not all case conferences have proven to be effective. The Norwegian Targeted Interdisciplinary Model for the Evaluation and Treatment of Neuropsychiatric Symptoms (TIME) is an effective multicomponent model based on case conferences that informs approaches to behavioural and psychological symptoms in residents with dementia in nursing homes. Our aim was to explore how TIME case conferences structured based on cognitive behavioural therapy (CBT) contributed to person-centred actions and how the specific inductive structure of the TIME may have contributed to the effectiveness of the model.

**Methods:**

We used video observation of six case conferences and analysed these videos by performing a thematic cross-case analysis of the transcripts from the videos and by iteratively watching the videos. According to Habermas’s theory of communicative action, we emphasized the case conference content, i.e., what was talked about in the case conferences, and the display of communication between the participants in the case conferences.

**Results:**

Our findings showed that the theoretical principles behind the TIME, including both person-centred care and the inductive structure of CBT, reflected many aspects of Habermas’s theory of communicative actions. In particular, the TIME case conferences emphasized the lifeworld perspective for both residents and staff and contributed to what Habermas labelled communicative rationality as a means to develop shared understanding among staff and create person-centred action.

**Conclusions:**

One causal assumption of how and why the TIME case conferences contributed to the effectiveness of the TIME in reducing BPSD in nursing homes is that the specific inductive structure of the case conferences with the column technique based on the ABC method together with PCC, emphasized the importance of the lifeworld for both the resident and the staff. Even though case conferences have been highlighted as useful, it is not indifferent how these case conferences are structured and conducted.

**Clinical trial registration:**

The trial TIME was registered January 6, 2016, with clinicaltrials.gov (NCT02655003).

## Background

In Norway, 84% of nursing home residents have dementia [1], and approximately 75% have clinically significant behavioural and psychological symptoms of dementia (BPSD), including agitation, aggression, anxiety, depression, psychosis, and apathy [2, 3]. These symptoms present challenges for patients themselves, their relatives, and the staff caring for them. In a randomized controlled trial, the use of the Targeted Interdisciplinary Model for Evaluation and Treatment of Neuropsychiatric Symptoms (TIME), based on case conferences, resulted in clinically significant reductions in agitation/aggression (primary outcome) and in symptoms of delusions, depression, and disinhibition, as well as improved quality of life (secondary outcomes) [4, 5]. In addition, among staff, the TIME shifted the way of learning from traditional to more innovative and reflection-based learning through a process of learning how to learn at work [6]. In the last report from the Lancet Commission from 2020 on dementia, prevention, intervention and care, the Norwegian TIME was one of two evidence-based approaches that were highlighted as effective multicomponent models for approaching agitation and aggression [4, 5, 7, 8]. The TIME was also mentioned as one of the nonpharmacological approaches with the largest effect sizes in a recent systematic review on pharmacological and nonpharmacological approaches to reduce disinhibited behaviours in dementia [9]. Case conferences have been used and recommended across different settings in health care to approach BPSD in residents with dementia [10, 11]. However, why some case conferences compare to others are effective as an approach to BPSD is poorly understood. The present study explored the case conference component of the TIME study. We used video observation to explore the communicative space that the case conferences in the TIME created in nursing homes. The use of video as a data source gave us unique access to explore the social interactions that occur in the case conference that would otherwise remain invisible to researchers. We searched the literature for theoretical concepts that reflected what was going on in the case conferences, which were characterized by interactions through multiple dialogues between the staff members. Using theory in qualitative research can serve as an expression of abstraction, and can enhance the ability to think across individual cases [12, 13]. Discovering Habermas’s theory of communicative action made our pieces come together [14, 15]. We will therefore use this theory to analyse our data, discuss our findings, interpret them and propose theoretical assumptions of why the specific structure of the case conferences in the TIME contributed to the effectiveness of the model. How and why does it work? A video-based qualitative analysis of case conferences to reduce BPSD through the lens of Habermas’s theory of communicative action.

### The targeted interdisciplinary model for evaluation and treatment of neuropsychiatric symptoms (TIME)

The TIME was developed in 2008–2009 in the field of practice at Tjærahågen Nursing Home in the municipality of Rana, Norway. It is based on several years of work with residents with dementia and additional symptoms that have created major challenges. The model was further developed at the Research Centre for Age-Related Functional Decline and Disease, Innlandet Hospital Trust [16]. The TIME represents a biopsychosocial approach and is a multicomponent manual-based intervention for the entire group of staff members caring for a resident as well as the physician and the ward leader. The intervention with the TIME consists of three phases that partly overlap. The first phase is the assessment phase. The second phase is the guided reflection phase, with case conferences, and the third and last phase is the action and evaluation phase [7]. The TIME is based on theoretical principles from both person-centred care (PCC) and cognitive behavioural therapy (CBT) [17–19].

From PCC, relational aspects are emphasized in the understanding of behaviour, and the approaches include biological, psychological and social aspects [19]. This means that a broad biopsychosocial assessment is required as part of the assessment phase. In the assessment phase, the resident’s individual history is also obtained, with a focus on the resident’s preferences, values and resources. This information is then taken to the case conference and presented there. In the case conference, one of the main questions that should be reflected on is the following: what is the resident’s perspective of the situation? The treatment measures that are developed in the case conference must be tailored to the individual resident [7, 16]. From CBT, the problem-solving method involving the use of the column technique on a whiteboard or a displayed screen, based on the ABC method, is adapted and used in the TIME case conference [20]. CBT emphasizes that our behaviour (C) and emotions (C) are the results of our beliefs (B) and interpretations (B) about an event (A). Every problem or situation is analysed in detail within the columns for (A) situation (facts), (B) thoughts (staff’s interpretation), (C) emotions (staff’s emotions and reactions) and (C) behaviour (treatment actions) [7]. In the nursing home, this means that our interpretation of a resident’s behaviour affects our own behaviour and emotional reactions towards the person. By changing our interpretations and actions towards the resident, we can contribute to maintaining, strengthening, or changing the behaviour of the resident [5, 6]. Both CBT and the TIME can be described as democratic methods with the goal of developing and using the participants’ (in the TIME, the staff’s) skills and resources rather than having to continually build on external experts. Socratic dialogue from CBT is used in the TIME case conferences. Socratic dialogue is a guide that helps participants use questions that are exploratory and promote logical thinking [16, 21]. The Socratic dialogue in the TIME case conference includes these aspects, as shown in Table 1.

**Table 1:** Socratic dialogue from CBT used in the TIME case conference [7, 16]

• Be empathic
• Ask open questions to encourage reflections
• Avoid direct leading questions
• Dig deeper
• Ask for justifications
• Determine whether there are other ways to understand what is happening
• Invite the participants to take residents’ perspective,
• Present a suggestion of interpretation if the group is stuck
• Always sum up

The case conference in the TIME starts with a short summary of what is known about the resident, including his or her personal history, with the main emphasis on the resident’s preferences and resources. After that, the medical history is referenced. Then, the staff create a list of problems that they consider important and prioritize among them. From the problem list, one problem at a time is discussed using the cognitive column technique [16, 18]. An overview of the structure and the recommended time used is presented in Table 2.

**Table 2:** Overview of the TIME case conference meeting [7, 16] ^1^ SMART: Specific, Measurable, Actual, Realistic, Time framed

Agenda for the case conference (60 min)				
**Activity**	**Preparation**: Convene a meeting and prepare a meeting room with a blackboard or similar facilities (projector, if available). Check that a flip pad and markers are available	TIME administrators: One is the chairman for the meeting. One takes notes on the whiteboard. One writes the minutes on the 5-column sheet.		**Responsible**
	**1. Status Report**: Personal history and main points from the patient’s medical record are presented.	10 min	Decide in advance, who should prepare and present the patient’s personal history and the main points from the medical record.	
	**2. Create a problem list**	5 min	Staff (as many as possible should attend the conference) The leading registered nurse and the nursing home physician should attend the conference, if possible.	
	**3. Prioritize problems from the list**			
	**4. Draw a 5-column sheet on the whiteboard**: facts – interpretations (thoughts) – emotions – actions – evaluation	40 min		
	**5. Describe facts from the registration and assessment phase**: one problem at a time			
	**6. Suggest interpretations** – guided discovery – discuss and reflect on them			
	**7. Describe any emotions experienced by the staff** – with interpretations by the staff			
	**8. Suggest SMART**^**1**^**actions** – based on the interpretations – decide how and when to perform **an evaluation** of the actions			
	**9. Summarize** interpretations and actions – close the meeting	5 min	TIME-administrator (chairman)	

### Case conferences: creating a communicative space in nursing homes

Case conferences are described as an approach that brings together relevant health and care professionals to discuss and agree on person-centred measures for the resident based on best available evidenced-based practice [11, 22]. In a systematic review by Reuther and colleagues, case conferences are described as a goal-oriented, systematic method that team members can use to exchange professional opinions on a particular care problem [10]. Due to the complex care needs of nursing home residents, the value of interdisciplinary care and the importance of PCC planning are reflected in various policies and standards [8, 23]. Case conferences often include different health professionals, such as ward leaders, physicians, registered nurses, auxiliary nurses and assistants, and can thereby facilitate interdisciplinary care in nursing homes [11]. The conference allows for sharing different perspectives regarding what the resident might want and gives the health care team an opportunity to discuss challenges and come to an agreement about the goals of care, which are important in creating person-centred measures for the resident [5, 11]. The results from two systematic reviews indicate that case conferences can reduce BPSD in nursing home residents [10, 11]. At the same time, other studies using case conferences have not shown reductions in BPSD [24–27]. A review from Reuther and colleagues highlights that the reflection process in case conferences should be oriented on a fixed sequence and role structure [10]. Focus group interviews with staff from the TIME study show that the structure of the case conference meeting with the use of the column technique from CBT was regarded as a factor that created creativity, new learning, security and coping [6]. However, how and why the communication process in TIME case conference meetings leads to person-centred measures and the effectiveness of the model have not been fully explored. In the present study, the communicative process within the case conference meeting in the TIME will be discussed with concepts from the theory of communicative action [14].

### The theory of communicative action by Habermas

Habermas’s theory of communicative action highlights the ongoing struggle between different rationalities [14]. The way things are marked, the meaning they establish, hold, change and the sense they reflect is affected by different rationalities, which in turn is the basis upon how communication is set. Based on this rational discussion, Habermas distinguishes between communication affected by the systemworld and communication affected by the lifeworld [14, 15]. He derives the terms “systemworld” from sociology and “lifeworld” from phenomenology. The systemworld is connected to the regulation of society, often affected by a result-oriented rationality directed towards individual or societal goal achievement. This result-oriented rationality is also labelled instrumental rationality and is dominated by creating means to an end. The lifeworld, on the other hand, comprises personal traditions, norms and cultural values and is essential to making decisions concerning individual human beings. However, Habermas is concerned that the result-oriented rationality influence of money and power in the systemworld has led to a society becoming increasingly divided into subsystems, with a growing impact of guidelines from economics and politics on our lifeworld. Thus, the following question can be posed: can the tension between the lifeworld and systemworld be seen in the daily meetings between staff and residents where communicative rationality can be challenged by instrumental rationality?

Habermas suggests that communicative rationality within a lifeworld perspective occurs when we use language to speak and express ourselves but at the same time direct ourselves towards others and establish relations with them. This in turn leads to an achievement of a greater insight into matters pertaining ourselves and the world around us with the use of language to justify statements and arguments, ask for reasons and answer questions [15, 28]. However, for the ideal speech situation to be realized, Habermas describes four phases of validity claiming that must exist. These four phases are truth, normative rightness, sincerity, and comprehensibility [28, 29]. The last claim, comprehensibility, is directly related to the use of language as a medium for communication. Hence, comprehensibility can be seen as a basis for the three other claims because if the speaker does not use a language that is understandable, no communication will take place. The other three claims, namely, truth, normative rightness, and sincerity, are associated with the three functions of language that Habermas presupposes: cognitive use, interactive use and expressive use [29]. These three functions are rooted in Habermas’s ontology, in which he divides reality into three worlds: the objective world, the social world and the subjective world. For a communicative process to occur where these validity claims are the base, participants must have an open attitude, seek to understand each other, and be willing to change their interpretation when new information and new insight are obtained [28, 30]. This is referred to as being moved by discovering the force of the better argumentation [31]. The validity claims and their relations to language, functions and domains of reality are described in Table 3.

**Table 3:** Validity claims and their relations, inspired by Habermas’s theory of communicative action [14, 15, 29]

Validity claims	Functions of language	Worlds (domains of reality)
Truth	Cognitive use	Objective world
Normative rightness	Interactive use	Social world
Sincerity	Expressive use	Subjective world

### Aims

Our aim is to explore how case conferences in the TIME structured according to CBT contribute to a specified communicative space resulting in person-centred actions and why this specific structure may contribute to the effectiveness of the model. According to Habermas’s theory of communicative action, we emphasize the content, i.e., what is talked about in the case conferences, and the display of communication among the participants.

## Design and methods

A qualitative explorative design based on the analysis of videos of case conferences in the TIME study was used. Videos in research provide extended opportunities for studying in detail the complexity of interactions and events that take place in social groups [32, 33]. Video differs from any other form of qualitative data because it provides a fine-grained multimodal record of any event, providing details on participants’ gaze, such as expressions, body language and gestures, as well as the atmosphere of the meeting. In addition, the use of video in this study gave us an opportunity to gain a deeper understanding of how staff use the structuring column technique from CBT to guide their communication with the aim of creating person-centred measures for the resident. This knowledge would have remained invisible to the researchers if only interviews or transcripts had been used. This study followed the Consolidated Criteria for Reporting Qualitative Research (COREQ) [34].

### Settings and participants

In the randomized controlled trial, 17 nursing homes received the TIME intervention. These nursing homes were located in the northern, central and south- eastern parts of Norway [4, 7]. In the present study, a convenience sample of six of the seventeen nursing homes that received the intervention was selected for the video recordings based on geographical proximity to the research centre. We video-recorded one case conference from each of these six nursing homes in the TIME intervention group. A total of 42 staff members participated in these six case conferences. The case conferences were composed as follows: two groups with five participants, one group with six participants, one group with seven participants, one group with eight participants and one group with eleven participants. The sex and profession of these 42 participants are presented in Table 4.

**Table 4:** Participants in the six case conferences (*n* = 42)

Background characteristics	Number (%)
**Sex**	
Female	39 (92,8)
Male	3 (7,2)
**Profession**	
Assistant	9 (21,4)
Auxiliary nurse	17 (40,4)
Registered Nurse	11 (26,2)
Leading ward nurse	2 (4,8)
Physician	3 (7,2)

### Data collection

The video recordings were performed by two researchers from the research team by setting up two small, fixed cameras in the room where the case conference took place. One camera was placed in the back of the room to capture the leader of the case conference and the shared display, and the other was placed in front to capture all the participants. The cameras were synchronised by a connection to a computer controlled by the research team. The members of the research team left the room after having started the cameras just before the starting point of the case conference. The staff contacted the research members to stop the cameras when the conference was over. The researchers did not observe the conference while it took place via the cameras or the computer and did not take part in the case conference. We did not want to interfere with their participation and wished to disturb the conferences as little as possible since they should be conducted by the staff alone, according to the study protocol for the TIME trial [7]. If one of the researchers was present at the case conference, such as for participant observation, we believed it would be difficult for the researcher not to interact with the staff members. Conducting case conferences was a new skill for the staff, and it would be natural for them to ask for advice on how to conduct the conference. The staff were not instructed to omit the name of the residents who were the subjects for the conference, as this would be difficult to control and might also distort the discussion during the conference. All six videos were transcribed verbatim, retaining pauses and emotional expressions.

### Data analysis

The analysis was performed by both authors simultaneously by a continuous iterative process involving systematic text condensation (STC) [35]. STC is a method for thematic cross case analysis. Inspired by Habermas’s theory of communicative action, we decided a priori to perform a combined deductive – inductive approach with the two main themes: (1) what was talked about and (2) how communication was displayed [14, 15, 30]. We decided to not take into account non-verbal signals or facial expression in our analysis, as we wanted to focus on the content and the display of the verbal dialogue. In addition, the placement of the two small, fixed cameras did not allow for capturing details of non-verbal communication by the participants. We followed four steps for the STC. (I) First, we watched the videos several times using the transcribed text to obtain an overall impression and to develop preliminary themes and emerging categories. (II) Next, we identified meaning units (i.e., dialogues) representing different aspects pertaining to the two main themes and developed code groups. The content of each meaning unit was condensed, and illustrating sequences of a discussion were identified. Since the main focus of this study was the content of the case conferences and the display of dialogues, sequences of discussion, rather than quotes, were identified. (III) Then, we created subgroups based on similarities and common semantic aspects of each code group. (IV) Finally, we summarized the condensation from each of the code groups into a general description and concepts reflecting the two main themes: what was talked about and how communication was displayed.

### Ethical consideration

The Norwegian Centre for Research Data (NSD) and The Regional Committee for Medical and Health Research Ethics in eastern Norway (REC South East) approved the study (Project No.: 2015/1549). Each participant signed a written consent to participate form after receiving oral and written information about the study. All identifiable characteristics were excluded from the presentation of data to ensure the anonymity of all participants and residents.

## Results

The characteristics of the participants are presented in Table 3. In three of the six case conferences, the physician was present, and in two meetings, the leading ward nurse was present. A shared display was used in all the case conferences visualizing the column technique. This display was either a blackboard or large sheets of paper attached to the wall. In all case conferences, this display was visible to all participants in the meeting. What the participants talked about was written down, and thus, this display oriented and guided the communication. Four of the case conferences were conducted with one of the staff leading the meeting and another staff member writing on the shared display. In two meetings, the same staff member was both leading the meeting and writing on the display. Within each of these two main themes, i.e., what was talked about and how the communication was displayed, our analysis revealed different categories, which are presented in Table 5.

**Table 5:** Themes and categories from the analysis

Themes	Categories
What was talked about	Who is the person?
	Agreement upon the main problem to be discussed and description of the facts
	Interpretation based on the person’s perspective
	Expression of their own feelings and putting into words their experienced daily dilemmas
	What actions to take?
How communication was displayed	The devil is in the details
	Rich argumentation on an equal level without the use of power
	Structured communication

## What was talked about

The main theme “What was talked about” consisted of five categories: (1) who is the person?, (2) agreement upon the main problem to be discussed and description of the facts, (3) interpretation based on the person’s perspective, (4) expression of their own feelings and putting into words their experienced daily dilemmas, and (5) what actions to take? Each of these categories will be described below.

### Who is the person?

Each of the case conferences started with a presentation of the resident, comprising his or her personal background history, including resources and preferences. This knowledge was then used throughout the case conference, both to better understand the problems at stake and to create person-centred actions to take. The participants used different examples from conversations they had with the resident and information from his or her background history when trying to interpret the problem at hand from the perspective of the resident. This can be seen in a dialogue from case conference video 3, quotation 1:

*1. This lady is actually used to sitting up until late at night before she goes to bed, and then she is so tired that she falls asleep and sleeps the rest of the night. Now she goes to bed early and sleeps until twelve and is awake the rest of the night. So, the question is, should she sleep from eight to twelve and be awake from twelve to eight, or should she sleep from twelve to eight?*

*2. Is that a habit of hers, to go to bed late at night?*

*1. Yes, for years.*

*2. Is this something we should try, the next fourteen days? To help her to bed at twelve?*

*3. Yes, I agree, if this is a habit of hers, then the night shift should help her to bed.*

### Agreement upon the main problem to be discussed and description of the facts

In all case conferences, a theme for the conversation was created by the staff through the development of a problem list. The problems were then sorted and numerated through a discussion, which resulted in an expressed agreement between the participants of which problem should be the theme for the case conference. Then, the objective facts about the chosen problem were described and presented on the shared display. Some of these facts originated from the results from the assessment phase. In all the case conferences, a presentation of facts pertaining to the problem chosen to be discussed took place. The validity criterion for something to be judged as a fact was that the participants in the conference believed that it was true and that it could be observed and measured (e.g., counted). This can be seen in a dialogue from the case conference in video 2, quotation 1:

*1. Before we move on, I think we have to take a look at the 24-hour behaviour and symptoms registration form to see what has happened last week*

*2. Shall we see….the, last eight days XX [name of the resident] has slept all nights, so that’s a fact*

*1. That is a fact, yes*

*2. Then, four of these days she has retired into the room, it’s a bit uneven how long she has been in the room, but five hours one day, one and a half, one and a half and three hours the other days. Then, there was a symptom of depression, which was noticed before noon and at noon every day*

*1. Mmm, yes, facts*

The facts that are described include knowledge about the resident’s situation, resources and challenges. The facts from the medical history and the assessment phase, including the residents’ personal history, are also used when the staff are trying to interpret the situation in the next column, the thoughts (interpretation) column.

### Interpretation based on the person’s perspective

In the column for interpretation, the staff tried to understand the causes of the problem they were discussing. In all videos, the staff gave different interpretations related to biological factors, psychological factors and social factors. The results of different assessments described in the column for facts were also used when the staff discussed different interpretations pertaining to the problem, for example, whether it could be related to pain, anxiety or depression. In addition, the background history of the resident was considered, and questions such as what the resident was accustomed to doing, what he or she would perceive as good or desirable today, or how the resident would interpret the situation were discussed. Interpretation of the situations from the perspective of the resident contributed to new knowledge of the situation and an expressed shared and common understanding of the resident’s reactions in some situations. This can be seen in a dialogue from video 5, quotation 1:

*1. How did she deal with personal hygiene before? Maybe she didn’t shower or wash herself every day?*

*2. Yes, is it possible that she has not been so careful with her appearance before?*

*3. I have actually worked for her before, and she was never very careful with her appearance but always washed like everybody else*

*4. Maybe she is just shy?*

*2. Yes, we can write that down in the column for interpretation*

*4. I would not have liked it either that a new person came into my room every morning*

*2. Maybe she also perceived it as a defeat to be dependent on help*

The discussion of the interpretations also revealed thought patterns from individual staff members, for example, wondering whether the resident was doing something on purpose. The videos revealed that the staff helped each other interpret this in a more appropriate way based on the facts and the residents’ perspectives. This can be illustrated in a dialogue from video 1, quotation 1:

*1. It is almost like she is doing it on purpose*

*2. Well, maybe we can look back to the facts then, she had an MMSE (Mini-Mental State Examination) last year of nine points. This test was not fully completed because she interrupted the assessment. She would probably have scored a little higher if she had completed the test, but her cognitive functions were clearly reduced. So, is it on purpose that she hid the medication, or can it be that she doesn’t understand that she needs to take it?*

*1. Yes, okay then*

*3. I think one interpretation of why she hides the medication should be that she has dementia. Maybe we should write that down?*

### Expression of their own feelings and putting into words their experienced daily dilemmas

Within the column for feelings, the videos revealed that the staff were given the opportunity to express the problem from their perspective. When the staff verbalized their feelings, their thoughts behind the feelings were also revealed, such as the dilemma of balancing the time spent on one resident with special needs with having enough time to support all the other residents. Their feelings and thoughts related to the situations where they perceived that they were not doing their job well enough were often discussed. One often-mentioned example in this regard was when a resident rejected care. When feelings were expressed, we observed in the videos that the staff supported each other, and in this way, they helped each other and sometimes change their thoughts behind difficult feelings. We observed this in a dialogue from video 1, quotation 2:

*1. Is it the fear of aggression from the resident, or is it…. Because, if we listen to what you say, I understand that you feel sorry for her [the resident] for not taking her painkillers*

*2. Yes*

*3. But it is so important that she take her medication, because if not, what can we expect?*

*1. Yes, there is a lot of expectation and thoughts about this…*

*2. And the feeling of not doing a good job. I feel it in my stomach when talking about this*

*1. But we are doing a good job, even if she doesn’t take her medication*

### What actions to take?

After the facts of the problems have been presented and interpreted and the staff had been given time to express their emotions in relation to the problems, the staff then started to discuss actions to take. The aforementioned interpretations and the resident’s background history were used to create actions that were tailored to the individual person based on who the person is and what he or she is used to. The staff also discussed resources and the feasibility of the suggested actions. Here, the dialogue often revealed a struggle between what the staff wished to do for the resident, based on what the staff knew the resident would have preferred, and the resources available. This is illustrated in a dialogue from video 5, quotation 2.

*1. If it is an action that she should shower in the evening, we must have enough staff at work*

*2. Yes, we must do it in the evenings when there are at least three staff members working*

The participants in the meetings also described how this way of reflecting together stimulated creativity and the creation of new ideas and new actions that they had not thought of before. We noted this in a dialogue from video 6, quotation 1.

*1. It is a little strange, because we just talked about it yesterday, what should we do, we have tried everything. Then, in this meeting, suddenly some new actions to take are coming up*

*2. We get trapped in our old habits*

*3. Yes, it is so wonderful that we, after three years with this resident, still can create new actions*

## How communication was displayed

The other main theme, “how communication was displayed”, consists of three categories: (1) the devil is in the details, (2) rich argumentation on an equal level without the use of power, and (3) structured communication. Each of these categories will be described below.

### The devil is in the details

A detailed description of the problem and the continuous questioning between the staff into every detail to find a feasible solution was evident in the videos. Very often, every detail in a complex situation was described and integrated into a whole story on how to interpret and create new actions customized to the resident. The staff expressed an intention to gain insight into the unique and unexpected aspects that were inherent in every situation, which was also unique for each individual resident. This discussion was often combined with general knowledge, which included knowledge on dementia and unique knowledge about the resident, to create solutions for the resident and an agreement on how the staff should meet the resident’s needs in the best possible way. This can be illustrated in a dialogue from video 3, quotation 2:

*1. When doing daily care, what do we do?*

*2. I have experienced that she needs breaks during the care*

*3. In what way? What do you mean?*

*2. Well, after you have removed her sweater, you take a break before you move on to the trousers*

*1. But where do we do it (daily care)?*

*2. In the morning, we do it in the bathroom, and in the evening, in her room*

*1. Is it different to do it in the bathroom compared to her room?*

*2. No, it seems like that does not matter, but if we use a changing coat that can have an impact*

### Rich argumentation on an equal level without the use of power

In all the videos, we observed that the participants addressed each other as equals during the dialogue. The assistants, the nurses, ward leaders and GPs all listened to each other in a respectful manner with no apparent use of power, and all participants were equally permitted to ask for reasoning in the dialogues. During the discussion, different arguments were listed on the shared display, and usually justifications were asked for these arguments. During the argumentation, differences in opinions within the staff group were identified. Through the argumentation process with clarifications and justifications, an expressed joint understanding was usually developed. An open attitude could be observed as some of the staff members changed their own points of view during the discussion when they obtained new insight through better arguments. This can be seen in a dialogue from video 2, quotation 2:

*1. She probably just wants attention*

*2. Or to be seen and heard*

*3. Attention? Is it so? She is very quiet, so maybe she is just easy to ignore?*

*2. But she is not someone that we ignore. New staff members often go to her because she is so easy to talk to. I think she gets a lot of attention*

*4. Is it wrong then? Should we erase it from the column?*

*2. Yes, I think so; “easy to ignore”, take that away*

*3. “Attention”, can we take that away too?*

*5. Yes, she does not demand attention*

*4. She does not demand anything*

*1. But who does not need attention?*

*5. I think she needs encouragement*

*1. Encouragement, yes that was a good description; write that down*

### Structured communication

The videos revealed that the use of the column technique helped the participants structure their communication. The participants had not had much training in the use of the TIME, but the column structure worked as an aid for knowing what to talk about and in which order. The videos also revealed that some of the staff members brought the TIME manual with them to the case conference, and during the meeting, they used the manual to follow the structure. This can be seen in a dialogue from video 6, quotation 2:

*1. I have to look in the manual so that I know what our next step is. Yes, we have to make a problem list. What do we perceive as the challenges for the resident? Just say what arises in your mind*

*2. Is this the registration of facts?*

*1. No, that is the next step. First, we have to make a problem list and then prioritize it*

## Discussion

Our findings show that the case conferences in the TIME structured according to CBT contributed to a specified communicative space resulting in person-centred actions. The overall impression is that the TIME emphasized the lifeword perspective for both staff and residents and that concepts from CBT in the TIME case conference contributed to what Habermas labelled communicative rationality [15, 28]. Here, we will discuss these findings in light of the theoretical principles behind the TIME, i.e., PCC [19] and CBT [17], and the theory of communicative action by Habermas [14, 15], and we will explore how theoretical concepts from the theory of communicative action are reflected in the cases conferences in the TIME.

### TIME emphasized the lifeworld perspective in nursing homes

Our findings showed that the use of the TIME emphasized the lifeworld perspective of both the residents and the staff in the nursing homes. Habermas’s description of the lifeworld corresponds with PCC, used in the TIME, where each individual’s lived life is of importance [16, 19]. The three lifeworld dimensions described by Habermas, namely, the objective, the social and the subjective ‘worlds’, correspond with the column technique, based on the ABC method from CBT that is adapted and used in TIME case conferences [7, 15, 20]. This can be inferred from Habermas’s description of the objective world as consisting of knowledge that can be judged objectively, defined as the facts of the problem [15]. In TIME case conferences, the columns for (A) situation (facts) contain the facts of the problem, such as objective knowledge, which correspond with the validity claim truth [28, 29]. Objective knowledge was visible in the findings when the staff presented the residents’ medical history, their description of the symptoms, the assessment from the assessment phase of TIME and the agreement upon the main problem to be discussed. As illustrated in the quoted dialogue from video 2, quotation 1, the validity criterion for something to be judged as a fact was that the participants in the conference believed that it was true and that it could be observed and preferably measured. Other characteristics about the case conferences that were highly visible in our findings were that the performance of these conferences was marked by the staff and the residents’ significant social and subjective worlds [15, 30].

The social world according to Habermas consists of moral practical knowledge, rules and norms that govern social interactions and is associated with the validity claim normative rightness. This was seen in our findings through staff discussions of who the resident was and what was right for the resident. This was mainly performed using the resident’s background history, with a focus on relations with the staff, the institutional context and family and friends. Discussing the social world can be seen in light of CBT in TIME case conferences and the columns for (B) thoughts (staff’s interpretation) and (C) behaviour (treatment actions) [7, 18]. In addition, PCC used in the TIME also emphasizes that the relationship perspective in human behaviour is crucial to understanding BPSD [7, 19]. As illustrated in the quoted dialogue from video 5, quotation 1, interpretation of the situations from the perspective of the resident contributed to new knowledge of the situation and an expressed understanding of the resident’s reactions and behaviour. The social world of the staff, such as the social norms and rules that governed the nursing homes and the care culture, was also revealed in our findings. As illustrated in the quoted dialogue from video 5, quotation 2, the struggle between what the staff wished to do for the resident based on what the staff knew the resident would have preferred and the resources available was discussed. This struggle can be seen in light of Habermas’s description of the systemworld’s place in social institutions, such as the nursing home [15, 36]. The care culture in nursing homes can adapt to expectations of effectiveness, economy-motivated prioritizing, and documentation of results, rituals, and routines from the systemworld [36, 37]. However, our findings revealed that the TIME case conferences contributed to a common understanding of the social world for both the resident and the staff, which is, according to Habermas, crucial for reaching a proper decision based on communicative rationality [15, 28, 30].

The third word, the subjective world, consists, according to Habermas, of our personal intention, thoughts and emotions [15]. Our subjective world is revealed through dialogues with others where we truthfully and sincerely express our inner lives, which correspond with the validity claim sincerity [28, 29]. We found that interpretation based on the person’s perspective showed how the staff tried to grasp how the resident perceived the problem and what was congruent with his or her intentions, emotions or wishes. This can be seen in light of principles from PCC used in the TIME, such as collecting the resident’s background history, values, preferences and remaining resources and in the case conference and asking the main question: what do staff believe is the resident’s thoughts behind the symptoms [7, 19]? Our findings also revealed that the staff’s subjective world was given space in the TIME case conferences. Principles from CBT, such as the column technique based on the ABC method, with a column for emotions (staff’s emotions and reactions) (C) helped the staff put into words and openly discuss their own emotions and their thoughts behind these emotions. This revealed core values and norms as often universalized principles governing the daily life in the nursing home, such as staff not doing a good job if the resident did not take her medication, as shown in the quoted dialogue from video 1, quotation 2.

Evidence from the TIME study has shown increased coping for staff facing complex problems, such as residents with BPSD, through a new creative, situated learning process [5, 6]. Situated learning is a reflection-based learning developed when different participants share different perspectives rooted in a local situation and context [38, 39]. In TIME case conferences, the structure based on the ABC method from CBT is based on the given situation, the context and the information collected in the registration and assessment phase and is not restricted to predefined indicators or themes. This implies that the structure with the ABC method used in the TIME case conference can be described as an inductive method rooted in the residents’ and staff’s lifeworld, in contrast to case conferences using predefined indicators, which can be described as a deductive method rooted in the systemworld [24–27]. In addition, in contrast to other models based on case conferences, TIME emphasises the importance of an interdisciplinary and a biopsychosocial approach.

### Communicative rationality and concepts from CBT in the case conference

Our findings suggest that the staff largely used Socratic dialogue in their discussions when interpreting the residents’ BPSD. As illustrated in the quoted dialogue from video 2, quotation 2, a Socratic dialogue seemed to display itself as an ongoing process during the TIME case conferences, guided by the column technique from CBT. Socratic dialogue allowed and encouraged justifications of arguments and allowed the best argument to triumph without the use of power, regardless of the position or profession of the participant. In CBT, this lack of use of power is labelled collaborative empiricism, where the therapist and the patient together explore and discuss the patient’s problems as equal participants in a conversation [17, 40]. In case conferences, collaborative empiricism implies that participants and those leading the conferences discuss the challenges on an equal basis, where the main task of the leaders of the conference is to secure the structure of the conference [16]. The lack of use of power is also a core principle in Habermas’s claims for communicative rationality to take place [14, 15]. Communicative rationality strives to achieve an open discussion where people are genuinely interested in listening to others and promoting their own views based on truth, normative rightness, and sincerity. These features are well illustrated in the dialogue quoted from video 2, quotation 2, in which the communication also resulted in a new understanding of what was at stake for the resident. This new understanding represents what we labelled situated knowledge about the resident and the situation earlier in our discussion.

It can, however, be argued that it is not possible to eliminate all forms of power when people interact with each other during a dialogue [30, 41]. Even if all the case conferences in our study ended in a personalized treatment plan that the staff agreed upon after using the principles of communicative rationality, we cannot be truly confident that some participants might have disagreed without expressing their views openly. Some participants might have been more skilled than others in expressing their opinions and justifying them. The presence of the leading ward nurse and the physician in the case conferences might also have had an impact on the other participants by virtue of their authority. Even if our results did not reveal any apparent use of power from some of the participants in the case conferences, we cannot totally rule out that this might have been the case in some situations. Such deliberated use of power might hinder a sincerely and truthful dialogue resulting in a forced consensus. According to Foucault, power is ubiquitous in all relations and cannot be suppressed or totally omitted [41]. For Habermas, however, dialogues based on the principles of communicative rationality are an ideal to strive for and not always possible to achieve [14, 15]. The structure of TIME case conferences encourages participants to elicit and describe in detail the interactions between the staff and the patient. As our findings show, this was observed as a common feature in the case conferences as a result of the use of the column technique based on the ABC method in CBT. This is well illustrated by the quoted dialogue from video 3, quotation 2, on how to proceed to succeed in removing the resident’s sweater and trousers as part of the evening care. Being concrete and focused on such details demands a dialogue based on truthful observations that are shared among the participants, not to force a view but to use detailed and truthful observations as a tool to obtain consensus on the care measures to take. In CBT, being concrete and describing the facts as truthfully as possible is very often used as a starting point in clinical consultations to be able to understand the more general problems for both the therapist and the patient [17, 40]. Establishing common concrete and detailed descriptions of the challenges at stake is consistent with what Habermas wants to achieve with communicative action, i.e., a consensus of the facts in the objective world as a starting point for interactions between the participants in a dialogue [14, 15].

As we have discussed, the performance of case conferences in the TIME adheres to a large extent to the principles of Habermas’s communicative rationality and his theory of communicative action. This might have contributed to the effectiveness of the case conferences and the TIME and the creation of new situated and shared knowledge and person-centred action plans for the residents in nursing homes, thereby reducing BPSD for residents, as shown in the effectiveness trial [4, 5]. The use of Socratic dialogue and the ABC method from CBT with a mainly inductive approach operationalize communicative rationality. These inductive communication methods distinguish TIME from most other nonpharmacological approaches to reduce disinhibited behaviours in dementia. This is a causal assumption and as such cannot be proven; there are other causal assumptions that can explain why an intervention in an RCT is effective or not. Process evaluations of complex interventions have demonstrated that management support and organizational factors are the main factors for the adherence of the staff and leaders in the implementation process and thereby the effectiveness of an intervention [42, 43]. In addition, the degree to which the intervention is flexible, is easy to implement and can be adapted to the local context is also an important issue [5, 44, 45]. The process evaluation of the intervention with TIME showed a high degree of flexibility in the adoption of the model to the local context; it was relatively easy to implement, and there was a high degree of adherence of the staff and leaders to the model and the implementation process [5].

### Strengths and limitations of the study

One of the advantages of using video in this study is that it gave us a unique multimodal record of any event, providing details on the participants’ gaze, such as expressions, body language and gestures, as well as the atmosphere in the meeting, and it allowed us to access a reality that otherwise would be hidden from researchers. We used two small, fixed cameras to record the case conferences. The participants in all case conferences told the researcher when taking down the cameras after the case conference that they had forgot about the cameras because of their small size. Nonetheless, the cameras in the room may have had an impact on the participants but less of an impact than from participant observation. The videos also gave us a different source of data than just text. This strengthens the credibility and trustworthiness of the study.

The authors of this study, JM and BL, are a nurse and a physician, respectively, as well as researchers; during the last years, we have been involved in both the development and conduct of the TIME study. We have both worked several years in nursing homes, but none of those nursing homes were involved in the TIME study. Our close involvement with the TIME model has both advantages and disadvantages. The advantage is that we have in-depth knowledge about the TIME model and its use in practice, which was important for the formation of the research question and the analysis of the videos. However, our close involvement with the TIME may also have influenced our analysis, causing a more positive judgement of the model and the conduct of the case conferences. To counterbalance this possible partiality, the STC method used to analyse our data is clearly described and can easily be verified by others, contributing to the transparency of our study. At the same time, no studies have previously conducted an in-depth analysis of the content of case conferences as they appear live from videos. The findings of this study can therefore contribute new knowledge about how case conferences should be structured and thereby enhance the transferability of the study.

## Conclusion

One causal assumption of how and why the TIME case conferences contributed to the effectiveness of the TIME in reducing BPSD in nursing homes is that the specific structure of the case conferences with the column technique based on the ABC method together with PCC emphasized the importance of the lifeworld for both the resident and the staff. In addition, this structure of the case conferences contributed to what Habermas labelled communicative rationality as a means to develop shared understanding among the staff and create person-centred actions. The TIME case conference can be described as an inductive method, as the problem in the given situation is discussed, and the description of the context and the information collected in the assessment phase pertaining to the actual problem are always treated as the starting point. Other types of case conferences that use predefined indicators can, in contrast, be described as more deductive methods, in which the systemworld will probably be given more space. This means that even though case conferences have been highlighted as useful, it is not indifferent how these case conferences are structured and conducted.

## Data Availability

The transcripts from the videos are available on reasonable request. The videos are not available due to ethical reasons.

## Abbreviations

BPSD: Behavioural and psychological symptoms of dementia
CBT: Cognitive behavioural therapy
COREQ: Consolidated Criteria for Reporting Qualitative Research
PCC: Person-centred care
STC: Systematic text condensation
TIME: Targeted Interdisciplinary Model for Evaluation and Treatment of Neuropsychiatric Symptoms
